# Development of a High-Efficiency Hairy Root Transformation System for Diverse Cowpea (*Vigna unguiculata*) Genotypes

**DOI:** 10.3390/plants15101560

**Published:** 2026-05-20

**Authors:** Shumeng Zhu, Xiaojia Su, Jialin Gao, Gege Hu, Zihan Xiao, Jiahui Mo, Hanrui Liu, Mengliang Niu, Huixia Zhao, Yike Qian, Nian Wang, Lei Pan

**Affiliations:** 1 Hubei Province Engineering Research Center of Legume Plants, School of Life Sciences, Jianghan University, Wuhan 430056, China; m13939367376@163.com (S.Z.); suxjia0805@163.com (X.S.);; 2 College of Horticulture and Forestry Sciences, Huazhong Agricultural University, Wuhan 430070, China

**Keywords:** cowpea (*Vigna unguiculata*), genetic transformation, hairy roots, *RUBY* gene

## Abstract

Cowpea (*Vigna unguiculata* (L.) Walp.) is a globally cultivated leguminous crop, but an efficient and stable genetic transformation system for cowpea is lacking. Thus, in this study, using different cowpea accessions, the main factors (genotype, explant, *Agrobacterium* strain for infection, and vector) affecting genetic transformation efficiency were systematically screened. Among the 43 cowpea accessions, two accessions (JD-0212 and A132) whose in vitro regeneration frequencies were high (propagation coefficient > 0.8 and adventitious bud induction index > 0.7) were identified. A system with a high infection rate for the two cowpea accessions was subsequently constructed, including cotyledonary nodes with cotyledons as the optimal explants, the *Agrobacterium rhizogenes* strain K599 and the *rbcs-RUBY* vector. Next, the system was optimized for its transformation conditions, such as infection duration, vacuum infiltration parameters and cocultivation time. The maximum transformation efficiency of genotype JD-0212 reached 82.79% under the optimal transformation conditions: 60 min of infection combined with 30 s of vacuum infiltration (−0.08 MPa), followed by four days of cocultivation. Furthermore, the transformation efficiency was validated in 86 cowpea accessions using two distinct vectors (*rbcs-RUBY* and *bs-EGFP*), indicating an average transformation efficiency of 42.09% (ranging between 4.04% and 82.79%). An efficient hairy root genetic transformation system for cowpea was established in this study.

## 1. Introduction

Cowpea (*Vigna unguiculata* (L.) Walp.), an annual herb of the genus *Vigna* of the Fabaceae family, is a globally cultivated edible legume crop [1]. Due to its high plant protein content and ability to improve soil fertility via symbiotic nitrogen fixation with rhizobia, cowpea plays a pivotal role in the development of sustainable agriculture and the safeguarding of food security [2]. However, an efficient and stable genetic transformation system for cowpea has not yet been established, hindering its functional genomic research and molecular breeding applications.

*Agrobacterium*-mediated whole-plant transformation systems, although widely used, have inherent limitations, such as a low transformation efficiency, strong genotypic dependence and a high chimerism rate [3]. In contrast, the Ri plasmid of *Agrobacterium rhizogenes* can integrate T-DNA fragments into the plant genome and induce the formation of hairy roots with profuse branching and rapid growth [4]. Due to its high transformation efficiency, short experimental cycle and straightforward operational procedure, the *A. rhizogenes*-mediated transformation system has been successfully applied. Although genetic transformation systems for nonleguminous crops [5,6] have been well established, their mature technical protocols are difficult to directly transfer to cowpea and other legumes because of substantial differences in the genetic background and physiological characteristics between species [7,8,9]. Moreover, the promoters and reporter genes commonly used in nonleguminous crops show weak compatibility with legumes and fail to efficiently drive the expression of target genes; thus, these genes are inadequate for overcoming the core bottlenecks in legume genetic transformation.

Research on genetic transformation systems in leguminous crops such as soybean (*Glycine max*), pea (*Pisum sativum*), and chickpea (*Cicer arietinum*) started at a relatively early stage [7,8,9]. Among these crops, the genetic transformation of soybean has been the most systematically and thoroughly investigated, and its transformation system has been successfully applied in cutting-edge fields, including CRISPR/Cas9-mediated precise gene editing [7]. However, this system still has obvious limitations and is characterized by a strong genotype dependence. Transformation efficiencies of the same protocol can differ by more than 10-fold among different soybean cultivars [7]. In addition, some widely adaptable elite varieties suffer from a low regeneration capacity and a high proportion of chimaeras. Similar constraints also exist in the genetic transformation systems of pea and chickpea [8,9].

However, a hairy root transformation system for cowpea has not yet been reported. Therefore, the present study aimed to establish an efficient genetic transformation system for the hairy roots of cowpea by identifying the optimal combination of multiple influencing factors, including genotype, explant type, vector, and *Agrobacterium* strain, for infection and transformation conditions. Furthermore, the stability of this established system was validated using 86 cowpea accessions. The results of this study provide critical technical support for the genetic transformation system required for fundamental molecular research in cowpea.

## 2. Results

### 2.1. Establishment of a Genetic Transformation System for Cowpea Hairy Roots

**Figure 1:**
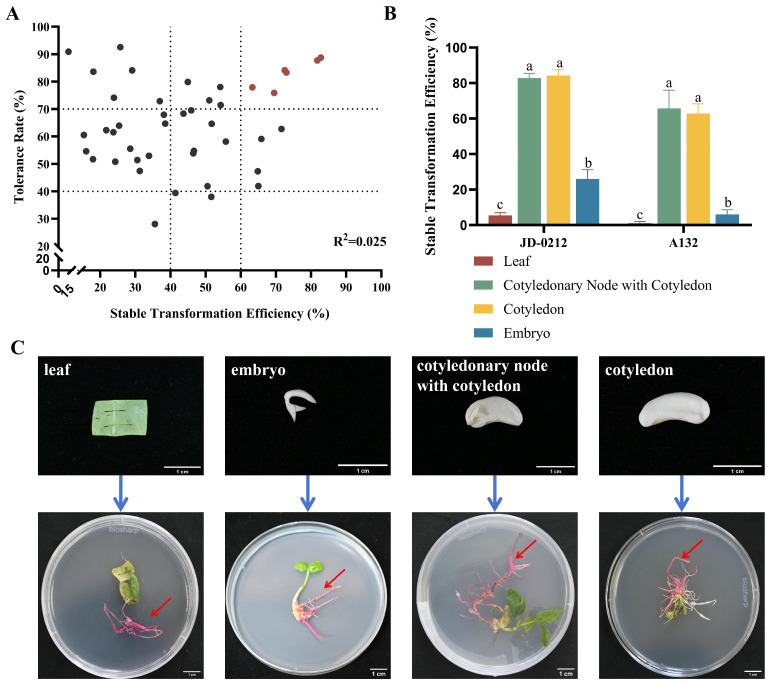
Establishment of a genetic transformation system for the hairy roots of cowpea. (**A**) The results of the screen 43 of cowpea accessions (Each dot represents two indicators of a single cowpea accession. Red dots indicate six cowpea accessions with tolerance rate above 70% and stable transformation efficiency over 60%.). (**B**) Stable transformation efficiency of the hairy roots of different cowpea explants (significant differences were determined using one-way analysis of variance (ANOVA) followed by Duncan’s multiple range test (*p* < 0.05)). (**C**) Phenotypes of hairy root induction from different cowpea explants; bar = 1 cm.

In this study, 43 cowpea accessions with high-frequency in vitro regeneration (a propagation coefficient > 0.8 and an adventitious bud induction index > 0.7) (Table S2) were evaluated based on the dual indicators of the strain tolerance rate and stable transformation efficiency. Six cowpea accessions with high tolerance and high stable transformation efficiency were initially screened (a strain tolerance rate > 70% and a stable transformation efficiency > 60%) (Figure 1A, Table S3). Further screening revealed two cowpea accessions (A132 and JD-0212) as highly compatible genotypes, whose stable hairy root transformation efficiencies reached 81.79% and 82.79%, respectively, and their tolerance rates to strain K599 were 87.71% and 88.73%, respectively—both of which were significantly greater than those of the other accessions (Figure 1A, Table S3).

These two accessions (A132 and JD-0212) were stably transformed to optimize the explant types, and significant differences in stable transformation efficiency were observed among the different tissue types: the hairy root induction rate of the leaf explants of JD-0212 was only 5.5%, and that of the embryonic explants was 25.93%, whereas the induction rates of the cotyledonary nodes with cotyledons and cotyledon explants reached 82.79% and 84.13%, respectively (Figure 1B). A132 displayed a similar pattern, with the same trend of a superior transformation efficiency in cotyledonary node and cotyledon explants (Figure 1B). The induction rate of cotyledon explants was slightly greater than that of cotyledonary nodes with cotyledons (Figure 1B).

**Figure 2:**
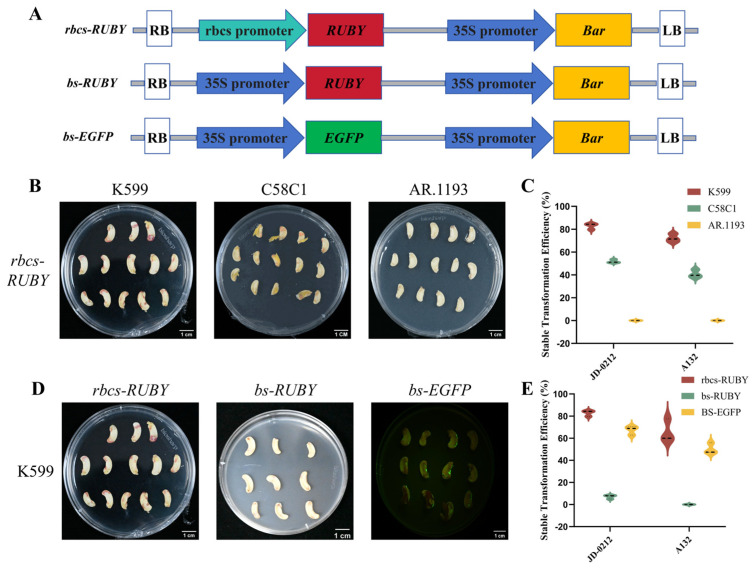
Optimization of vectors and strains for cowpea hairy root transformation. (**A**) Structural maps of the *rbcs-RUBY* vector, *bs-RUBY* vector, and *bs-EGFP* vector. (Arrows represent promoters, and rectangles indicate genes. Different colors distinguish distinct promoters and genes). (**B**) Phenotypes of different strains after transient transformation; bar = 1 cm (Red indicates the *RUBY* phenotype). (**C**) Efficiencies of different strains in the stable transformation of hairy roots. (**D**) Phenotypes observed after transient transformation with different vectors; bar = 1 cm (Red indicates the *RUBY* phenotype, and green fluorescence represents the *GFP* phenotype). Note: The same control image was obtained from parallel experiments under identical experimental conditions. (**E**) Efficiencies of different vectors in the stable transformation of hairy roots.

The two accessions (A132 and JD-0212) were transformed to screen vector–strain combinations. These two accessions combined with *Agrobacterium rhizogenes* strain K599 and the *rbcs-RUBY* vector resulted in high hairy root transformation efficiencies of 65.68% for A132 and 82.79% for JD-0212 (Figure 2C,E). In the present study, the efficiency of the expression of the *RUBY* reporter gene driven by the *rbcs* promoter in cowpea hairy roots was significantly higher than that driven by the *bs* promoter (Figure 2E), with the stable transformation efficiency being approximately 11-fold higher in the latter. In terms of transient transformation, the *rbcs-RUBY* vector showed the best transformation effect, followed by the *bs-EGFP* vector, and the *bs-RUBY* vector showed the worst transformation effect (Figure 2D).

### 2.2. Optimization of the Hairy Root Genetic Transformation System for Cowpea

**Figure 3:**
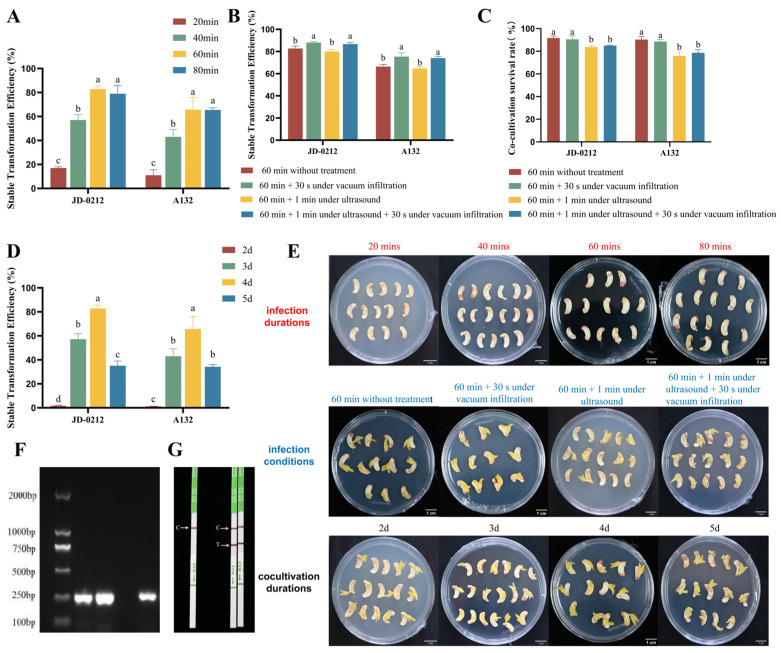
Optimization of the genetic transformation system for the hairy roots of cowpea. (**A**) Stable transformation efficiency of hairy roots at different infection durations (*p* < 0.05). (**B**) Stable transformation efficiency of hairy roots under different infection conditions (*p* < 0.05). (**C**) Survival rates of explants after cocultivation under different infection conditions (*p* < 0.05). (**D**) Stable transformation efficiency of hairy roots on different cocultivation days (*p* < 0.05). (**E**) Phenotypes observed after transient transformation with different infection durations, under different infection conditions, and for different cocultivation days; bar = 1 cm. (**F**) PCR verification of positive hairy roots (from left to right: marker, positive root 1, positive root 2, negative control, and plasmid control). (**G**) The results of PAT/bar colloidal gold test strip detection of positive hairy roots. Note: The same control image was obtained from parallel experiments under identical experimental conditions. Significant differences were determined using one-way analysis of variance (ANOVA) followed by Duncan’s multiple range test (*p* < 0.05).

The infection and cocultivation conditions were further optimized in this study to establish an efficient and stable hairy root genetic transformation system for cowpea. The results showed that both 60 and 80 min of *Agrobacterium* infection significantly improved the transient transformation efficiency and stable transformation efficiency (Figure 3A,E). Vacuum and ultrasonication were introduced as assisted treatments based on 60 min of infection to further increase the stable transformation efficiency. The effects of different treatment combinations on the transformation system were evaluated by integrating the stable transformation efficiency and explant tolerance rate as key indicators. The results demonstrated that the combination of 60-min infection + 30-s vacuum infiltration was the optimal treatment condition, under which the explants maintained a higher survival rate after cocultivation, and the transformation efficiency increased by 5.3% for A132 and 9.0% for JD-0212 compared with the control group without assisted treatments. Although the transformation efficiency increased in the ultrasonic treatment groups to a certain extent (Figure 3B), the survival rate of the explants decreased to varying degrees after cocultivation (Figure 3C), and the increased in transformation efficiency was significantly lower in the ultrasonic treatment group than in the vacuum infiltration combination group (Figure 3B).

The results of the optimization of the cocultivation duration indicated that a period of four days was the optimal duration (Figure 3D). This duration not only prevented the low transformation efficiency of positive roots caused by overly short cocultivation periods (<3 d) but also prevented the excessive proliferation of *Agrobacterium* and necrosis of explants induced by an overly long cocultivation duration (>5 d) (Figure 3D,E).

A combined detection method involving a PCR-based molecular assay for the *bar* gene and a transgenic PAT/bar colloidal gold test strip assay for the bar protein was used to verify the positive rate and gene integration status of cowpea hairy roots obtained under the optimal parameters. The PCR results revealed that a specific *bar* gene fragment (244 bp) could be amplified using genomic DNA isolated from the transformed hairy roots as the template (Figure 3F). The results of the transgenic PAT/bar colloidal gold test strip assay indicated that the homogenate of positive hairy roots produced distinct double bands on the test strip, whereas only the quality control band appeared in the negative control (Figure 3G). These findings confirmed that the *bar* gene was successfully expressed at the protein level and further verified that the optimized transformation system could efficiently generate positive hairy roots, with the transformation efficiency of positive roots reaching 82.79% (Table S3).

### 2.3. Validation of the Stability of the Hairy Root Genetic Transformation System for Cowpea

**Figure 4:**
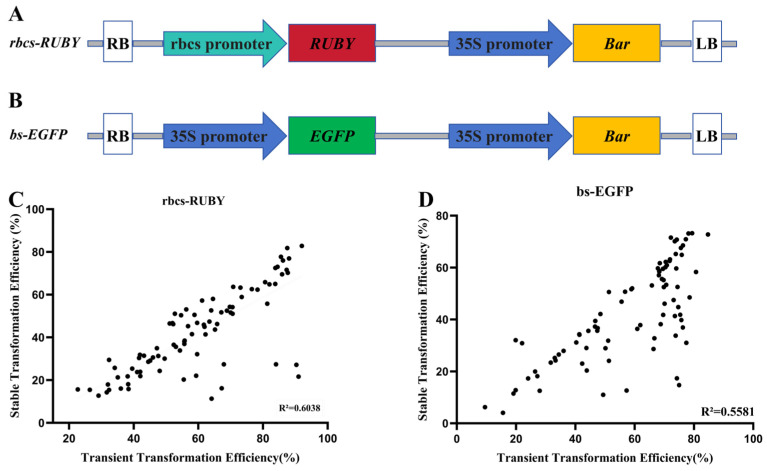
Verification of the stability of the dual-vector system for the hairy root genetic transformation of cowpea. (**A**) Structural map of the *rbcs-RUBY* vector. (**B**) Structural map of the *bs-EGFP* vector (Arrows represent promoters, and rectangles indicate genes. Different colors distinguish distinct promoters and genes). (**C**) Analysis of the correlation between the transient transformation efficiency and stable transformation efficiency of the *rbcs-RUBY* vector (*p* < 0.01). (**D**) Analysis of the correlation between the transient transformation efficiency and stable transformation efficiency of the *bs-EGFP* vector (*p* < 0.01).

In this study, genetic transformation assays were performed on 86 cowpea accessions to validate the stability and feasibility of the established hairy root transformation system using two vectors (Table S5), namely, *rbcs-RUBY* (Figure 4A) and *bs-EGFP* (Figure 4B). Based on the validation data from all 86 accessions, the *rbcs-RUBY* vector was identified as the optimal transformation vector for most cowpea accessions. Among these accessions, 59 exhibited a transient transformation efficiency of more than 50% with this vector (Table S4), and a significant positive correlation was observed between the transient transformation efficiency and stable transformation efficiency (Figure 4C). A similar positive correlation trend was also found for the *bs-EGFP* vector (Figure 3D). These results indicated that the infection, selection and transformation procedures of this system were coherent and efficient and that transient transformation efficiency could serve as a reliable predictive indicator of the efficacy of stable transformation (Table S4).

For the 86 cowpea accessions, the average stable transformation efficiency with the *rbcs-RUBY* vector was 41.95% (ranging from 11.32–82.79%), whereas that with the *bs-EGFP* vector was 42.23% (ranging from 4.04–73.26%). Although the average stable transformation efficiencies of the two vectors were highly comparable, considering the visual convenience of transformation screening and practical application value, the *rbcs-RUBY* vector is more suitable as the preferred tool for cowpea hairy root genetic transformation (Figure 2D, Table S2).

## 3. Discussion

In this study, an efficient and stable hairy root genetic transformation system was successfully established for cowpea by systematic optimization of genotype, explant type, *Agrobacterium* strain, vector, and transformation conditions. The system achieved a maximum stable transformation efficiency of 82.79% and was validated across 86 diverse cowpea accessions, representing a significant technical advance for this economically important but recalcitrant legume crop.

### 3.1. Genotype Dependence in Hairy Root Transformation

In previous studies on hairy root genetic transformation in legume crops, genotypic dependency restricted the universality of the transformation systems. This problem has been confirmed in *G*. max, *C*. arietinum, *P*. sativum and other legumes [10]. The dedifferentiation potential of plant cells determines the transformation compatibility of various accessions, and the enhanced callus induction capacity mediated by this potential dedifferentiation can synergistically increase transformation efficiency and strain tolerance [11]. Although the induction rate of cotyledon explants was slightly greater than that of cotyledonary nodes with cotyledons (Figure 1B), further analysis revealed that the cotyledonary nodes with cotyledons of A132 and JD-0212 presented advantages in terms of both a high stable transformation efficiency and a low browning rate in explants. The superior performance of cotyledonary nodes with cotyledons may be attributed to the vigorous division of meristematic cells in the junction region of cotyledons and hypocotyls [12]. Similar studies have reported that the true leaf primordia of common bean significantly inhibit explant browning [13].

Beyond genotype and explant type, the choice of vector–strain combination also played a critical role. The combination of *Agrobacterium rhizogenes* strain K599 with the *rbcs-RUBY* vector yielded the highest transformation efficiencies, significantly outperforming other combinations (Figure 2C,E). This result may be attributed to the high expression of the vir gene in the Ri plasmid of strain K599 [14]. Moreover, the *rbcs* promoter drove *RUBY* expression approximately 11-fold more efficiently than the bs promoter in cowpea hairy roots (Figure 2E), highlighting the importance of promoter selection for optimal transgene expression.

In summary, through genotypic screening, explant optimization and vector–strain combination screening, an efficient and stable hairy root genetic transformation system for cowpea was established in this study. Among the optimized factors, two cowpea accessions (JD-0212 and A132) were shown to have a high stable transformation efficiency, cotyledonary nodes with cotyledons were the optimal explants, and the combination of strain K599 and the *rbcs-RUBY* vector significantly improved the efficient stable transformation of positive roots.

### 3.2. Optimization of Infection and Cocultivation Parameters

Extending the duration of *Agrobacterium* infection could increase the contact frequency between the strain and the wounded cells of explants [15]. However, an 80-min infection tended to induce the excessive proliferation of *Agrobacterium*, which in turn led to adverse effects such as browning and necrosis. These adverse effects may be attributed to the damage to explant cell membrane integrity caused by long-term infection, which activates the polyphenol oxidase system; moreover, excess *Agrobacterium* competes for nutrients and secretes toxic metabolites [16]. Similar optimized screening methods for determining the optimal infection duration have been widely adopted in studies on other transient or stable transformation systems in plants [17,18]. In these studies, the optimal infection duration was also determined by balancing transformation efficiency and explant damage [17,19], a conclusion that is highly consistent with the results obtained in this study on cowpea.

To further enhance transformation efficiency, vacuum infiltration and ultrasonic treatment were introduced as assisted treatments. Vacuum infiltration can create a negative pressure environment to promote the sufficient penetration of *Agrobacterium* into the intercellular spaces of wounded explant cells, thereby increasing the contact probability between the *Agrobacterium* strain and target cells [20]. In contrast, ultrasonic treatment relies on microjets and shock waves generated by cavitation effects to slightly damage the surface cells of explants and expand the wound area; moreover, ultrasonic treatment accelerates the dispersion of *Agrobacterium* in the infection solution, thereby creating more favourable conditions for the strain to infect target cells [21]. Compared with the parameters reported in existing studies on hairy root transformation in other legume crops, the optimal transformation parameters determined in this study exhibited species-specific differences. For example, the optimal infection time for hairy root transformation of *P. sativum* was 30 min [8], and the optimal cocultivation time for *Lens culinaris* was three days [11].

In this study, the *rbcs-RUBY* vector and *Agrobacterium* strain K599 were used. This combination differs from systems commonly used in leguminous crops [22,23,24]. It was chosen primarily based on comprehensive considerations of strain virulence compatibility and vector suitability. Different leguminous plants exhibit significant differences in their responses to various *Agrobacterium* strains; the adaptability of the strain is directly related to the effective transfer and stable transformation of T-DNA during the infection process and affects the expression level of the reporter gene, thereby preventing misjudgment caused by weak or abnormal signals [23,24,25]. These species-specific differences arise from the variations in structural characteristics, cell wall components and *Agrobacterium* tolerance of explants among different legume crops, further highlighting the necessity of optimizing species-specific transformation conditions for cowpea.

### 3.3. Regulatory Role of Promoter Characteristics in Transformation Efficiency

Promoter characteristics are core factors that regulate transformation efficiency [26]. The *rbcs* promoter efficiently drives the expression of reporter genes in plant cells [27,28], and its high efficiency stems from the synergistic activation of *cis*-regulatory elements (e.g., G-boxes and light-responsive motifs) [29] and the specific binding of transcription factors such as GLK [30].

In addition to light-responsive elements, the promoters of some isoforms in the *RBCS* gene family also contain nonlight-regulated cis-elements, which confer basal transcriptional activity in roots [31]. Thus, gene expression driven by the rbcs promoter is more characteristic of a tissue preference than absolute tissue specificity [32]. Although roots do not perform efficient photosynthesis, they still maintain basic photosynthesis-related metabolism and plastid developmental requirements, leading to detectable promoter activity in nonphotosynthetic tissues such as roots [33]. Studies of chickpea (*Cicer arietinum*) have revealed that the strong expression and activity of the *rbcs* promoter are evolutionarily conserved among legumes [27]. In research on insect-resistant transgenic pigeon pea (*Cajanus cajan*), the *rbcs* promoter can efficiently drive the expression of insect resistance genes, further verifying its compatibility with legume hosts and its role as a key factor in regulating transformation efficiency [34]. In contrast, the *bs* promoter is of bacterial origin, and even after artificial optimization, it remains a host-specific restriction factor, leading to low transcription efficiency of downstream genes and insufficient expression of reporter genes, thus reducing screening efficiency and stable transformation efficiency [35,36]. In this study, the transformation efficiency was significantly improved (reaching 50.16–67.31%) when the *bs* promoter was combined with the *EGFP* reporter gene, suggesting a potential sequence compatibility issue between the *bs* promoter and *RUBY*. The expression of the *RUBY* gene relies on the synergistic effect of betalain synthesis and metabolic pathways in host cells, whereas *EGFP* is an independently expressed fluorescent protein that does not require the assistance of the host metabolic network [37]. These findings indicated that the expression of the *RUBY* gene is more compatible with the transcription signals of the promoter, whereas the sequence characteristics of *EGFP* are more compatible with the transcriptional output of the *bs* promoter. Thus, the differences in transformation efficiency between the two vectors may be closely associated with the origin characteristics and host compatibility of the promoters.

### 3.4. Limitations and Future Perspectives

Although the cowpea hairy root transformation system established in this study is efficient and broadly applicable, several limitations remain and warrant further optimization in future research [4]. The transformation efficiency of the 86 accessions was stable, with only a small number of genotypes showing poor compatibility. Future studies could integrate multiomics analyses to elucidate the molecular mechanisms underlying genotypic differences and employ genome editing to modify genes associated with *Agrobacterium* infection and plant defence responses, with the aim of developing a more genotype-independent transformation system [10]. The current protocol generates only hairy roots rather than intact transgenic plants, limiting its application in the study of above-ground traits [4]. Future work should focus on optimizing the shoot regeneration capacity and vector compatibility in cowpea, refining the genetic transformation system, and providing technical support for the targeted improvement of agronomic traits and molecular breeding in cowpea at the whole-plant level [3]. This operation is labour intensive and relies on strict aseptic conditions, limiting its high-throughput application [4]. Further simplification of the sterilization and culture steps and the development of nonaseptic induction methods will lower the technical threshold and improve experimental scalability [22]. Addressing these limitations will facilitate the establishment of a more universal, efficient, and multifunctional platform, thereby strongly advancing cowpea functional genomics, gene editing, and molecular breeding.

## 4. Materials and Methods

### 4.1. Plant Materials

A total of 43 cowpea accessions with a high in vitro regeneration capacity were used for genotype screening and transformation system optimization. Eighty-six cowpea accessions were employed as the comprehensive experimental materials, among which the aforementioned 43 regenerable accessions were included as a subset. All 86 cowpea accessions were obtained from Hubei Province Engineering Research Center for Legume Plants and Hubei Province Natural Science and Technology Resource Center for Edible Legumes, Jianghan University, Wuhan, China.

### 4.2. Bacterial Strains and Vectors

Competent *Agrobacterium rhizogenes* cells (strains K599, C58C1 and AR1193) were purchased from Weidi Biotechnology Co., Ltd. (Shanghai, China). The vectors *rbcs-RUBY*, *bs-RUBY* and *bs-EGFP*, all of which contained the target bar gene, were provided by Boyuan Biotechnology Co., Ltd. (Wuhan, China).

### 4.3. Experimental Methods

#### 4.3.1. Method for Inducing Cowpea Hairy Root Infection

Viable cowpea seeds with intact seed coats and a uniform size were selected. First, the seeds were rinsed with 75% ethanol for 30 s, followed by 2–3 washes with sterile water. The seeds were subsequently immersed in a 20% Kao detergent bleach (NaClO) solution and placed on a shaker (150 rpm, 20 min). After the bleach solution was discarded, the seeds were rinsed 5–8 times with sterile water until the water was clear. The seed surfaces were dried with sterile filter paper. The seeds were incubated for germination on water agar medium (Table S1) with the hilum facing downwards, overnight in the dark (26 °C, 12 h). A scalpel was used to cut along the midline of the cotyledons in each germinated seed. The primary radicle was removed while the primary leaf buds were retained, and slight scratches were made on the seed surface with a blade at the junction of the cotyledons and hypocotyls. The prepared explants were subsequently inoculated with resuspended *Agrobacterium rhizogenes* solution (referred to as the “infection solution”) (OD_600_ = 0.6–0.7) (Table S1), subjected to vacuum infiltration (−0.08 MPa) for 30 s, and then incubated on a shaker (50 rpm, 60 min) for infection. After infection, the resuspension liquid was blotted dry with sterile filter paper. The explants with cotyledons were incubated on a cocultivation medium (Table S1) with the wounded surface of the cotyledon nodes inverted upwards and then cocultured in the dark (23 °C, 4 d). At the end of the cocultivation treatments, the explants were transferred to 500 mg/L cefotaxime (Cef) solution (1 min) and rinsed 2–3 times until the solution was clear. After being rinsed with sterile water and dried with filter paper, the explants were inserted obliquely into recovery medium (Table S1), with the wounded surface facing downwards, and cultured at 26 °C for 4 days (16 h light/8 h dark).

Afterwards, the explants were incubated on Selection Medium I (Table S1) with the wounded surface facing downwards and transferred to fresh medium every 7 days. After 14 days, the explants were transferred to Selection Medium II with a relatively high Basta concentration (4 mg/L) for further culture (Table S1).

#### 4.3.2. Optimization of the Genetic Transformation Conditions for Cowpea Hairy Roots

The strain tolerance rate, transformation efficiency, explant browning rate and survival rate were adopted as evaluation indices to progressively optimize the genetic transformation conditions for cowpea hairy roots.

##### Genotypic Screen

For genotypic screening, cotyledonary nodes with cotyledons were used as explants. A fixed combination of *Agrobacterium* strain K599 and the *rbcs*-*RUBY* vector was used, with an infection time of 60 min and a cocultivation duration of 4 d set as the fixed conditions. A total of 43 cowpea accessions with high-frequency in vitro regeneration were subjected to genetic transformation. For each genotype, 100 explants were treated in three biological replicates to screen elite accessions.

##### Explant Type Screen

The elite accessions obtained from the aforementioned screen were used as the experimental materials, with the above strain–vector combination and fixed infection and cocultivation conditions unchanged. Four types of explants—leaves, embryos, cotyledons and cotyledonary nodes with cotyledons—were prepared. For each explant type, 100 explants were treated in three biological replicates to determine the optimal explant type.

##### Screening of Vector–Strain Combinations

The elite accessions obtained previously were used as the materials for separate screens of the vectors and strains. For vector screening, strain K599 was combined with three vectors, namely, *rbcs*-*RUBY*, *bs*-*RUBY* and *bs*-*EGFP*; for strain screening, the *rbcs*-*RUBY* vector was combined with three strains, namely, K599, C58C1 and AR1193. For each combination, 100 explants were treated in three biological replicates to determine the optimal vector–strain combination.

##### Optimization of the Transformation Conditions

Based on the optimal combinations of genotype, explant type, vector and strain obtained using the methods described above, gradients of were variables further set to optimize the key transformation conditions. The infection time was set at four gradients: 20, 40, 60 and 80 min. The infection treatment conditions were divided into four groups: 60 min (as a control), 60 min + 30 s of vacuum infiltration (−0.08 MPa), 60 min + 1 min of ultrasonic treatment (100 W, 40 kHz), and 60 min + 30 s of vacuum infiltration (−0.08 MPa) combined with 1 min of ultrasonic treatment (100 W, 40 kHz). The cultivation time was set at four gradients: 2, 3, 4 and 5 d. In each treatment, 60 explants were processed in three biological replicates. The optimal transformation conditions were determined by statistically analysing all the evaluation indices listed above.

#### 4.3.3. Identification of Positive Roots

##### Polymerase Chain Reaction (PCR)-Based Molecular Detection

Positive hairy roots (3–4 cm) with *RUBY* and *EGFP* phenotypes were quickly frozen in liquid nitrogen and ground into powder, after which the genomic DNA was extracted (TIANGEN Plant Genomic DNA Extraction Kit, Beijing, China). The purity and concentration of DNA were determined by measuring the OD_260_/OD_280_ ratio. PCR amplification was performed with the extracted DNA as the template, and the total PCR volume was 20 μL, containing 1 μL of DNA template (100 ng/μL), 1 μL of *Bar-F* (10 μM), 1 μL of *Bar-R* (10 μM), 10 μL of 2 × *E-Taq* PCR Master Mix (Yinmu Biotechnology Co., Ltd. Wuhan, China), and 7 μL of ddH_2_O.

The PCR protocol was as follows: predenaturation at 95 °C for 5 min, followed by 30 cycles of denaturation at 95 °C for 50 s, annealing at 63 °C for 50 s, and extension at 72 °C for 30 s. A final extension was conducted at 72 °C for 5 min, and the products were stored at 4 °C for subsequent use. The primers used were as follows: *Bar-F*: 5′-TGGGCAGCCCGATGACAGCGACCAC-3′ and *Bar-R*: 5′-ACCGAGCCGCAGGAACCGCAGGAGT-3′ (synthesized by Tsingke Biotechnology, Beijing, China) (expected amplicon size: 244 bp). The PCR amplification products were subjected to electrophoresis on a 2% agarose gel (120 V, 35 min), after which they were detected by a gel imaging system (Analytik Jena, Germany).

##### Transgenic Phosphinothricin Acetyltransferase (PAT)/Bar Colloidal Gold Test Strip Detection

Positive hairy roots (3–4 cm) with *RUBY* and *EGFP* phenotypes were placed in an extraction tube and ground with a grinding rod to produce a homogenate. After 0.4 mL of extraction buffer was added, the mixture was vortexed for 30 s to ensure uniform mixing. The MAX end of the test strip was then vertically immersed into the sample solution (ensuring that the liquid level did not exceed the MAX line). After reacting for 5–10 min, the strip was placed horizontally for the interpretation of the results, and the results were considered invalid if the time was exceeded. The criteria for interpreting the results were as follows: if both the control line (C) and the test line (T) developed colour, the results were considered positive; if only the control line (C) developed colour, the results were considered negative; and no colour development at the control line (C) was deemed invalid and required retesting.

### 4.4. Data Statistics and Analysis


$$\mathrm{Strain}\;\mathrm{tolerance}\;\mathrm{rate}\;(\%)=\frac{\mathrm{total}\;\mathrm{number}\;\mathrm{of}\;\mathrm{nonbrowned}\;\mathrm{explants}}{\mathrm{total}\;\mathrm{number}\;\mathrm{of}\;\mathrm{infected}\;\mathrm{explants}}\times 100$$

$$\mathrm{Transformation}\;\mathrm{rate}\;(\%)=\frac{\mathrm{total}\;\mathrm{number}\;\mathrm{of}\;\mathrm{explants}\;\mathrm{with}\;\mathrm{positive}\;\mathrm{roots}\;\mathrm{formed}\;\mathrm{by}\;\mathrm{transformation}}{\mathrm{total}\;\mathrm{number}\;\mathrm{of}\;\mathrm{infected}\;\mathrm{explants}}\times 100$$

$$\mathrm{Transient}\;\mathrm{transformation}\;\mathrm{rate}\;(\%)=\frac{\mathrm{total}\;\mathrm{number}\;\mathrm{of}\;\mathrm{transient}\;\mathrm{positive}\;\mathrm{explants}\;\mathrm{after}\;\mathrm{cocultivation}}{\mathrm{total}\;\mathrm{number}\;\mathrm{of}\;\mathrm{infected}\;\mathrm{explants}}\times 100$$

$$\mathrm{Survival}\;\mathrm{rate}\;\mathrm{after}\;\mathrm{cocultivation}\;(\%)=\frac{\;\mathrm{number}\;\mathrm{of}\;\mathrm{viable}\;\mathrm{explants}\;\mathrm{after}\;\mathrm{cocultivation}}{\mathrm{total}\;\mathrm{number}\;\mathrm{of}\;\mathrm{infected}\;\mathrm{explants}}\times 100$$

$$\mathrm{Proliferation}\;\mathrm{coefficient}=\frac{\;\mathrm{number}\;\mathrm{of}\;\mathrm{explants}\;\mathrm{with}\;a\;\mathrm{single}\;\mathrm{adventitious}\;\mathrm{bud}+\mathrm{number}\;\mathrm{of}\;\mathrm{explants}\;\mathrm{with}\;\mathrm{multiple}\;\mathrm{adventitious}\;\mathrm{buds}\;\times \;2}{\mathrm{total}\;\mathrm{number}\;\mathrm{of}\;\mathrm{explants}}$$

$$\mathrm{Adventitious}\;\mathrm{bud}\;\mathrm{germination}\;\mathrm{index}=\frac{\;\mathrm{number}\;\mathrm{of}\;\mathrm{explants}\;\mathrm{with}\;a\;\mathrm{single}\;\mathrm{adventitious}\;\mathrm{bud}+\mathrm{number}\;\mathrm{of}\;\mathrm{explants}\;\mathrm{with}\;\mathrm{multiple}\;\mathrm{adventitious}\;\mathrm{buds}}{\mathrm{total}\;\mathrm{number}\;\mathrm{of}\;\mathrm{inoculated}\;\mathrm{seeds}}$$

Transformation rate = (total number of explants with positive roots formed by transformation/total number of infected explants) × 100%.

Strain tolerance rate = (total number of nonbrowned explants/total number of infected explants) × 100%.

Transient transformation rate = (total number of transient positive explants after cocultivation/total number of infected explants) × 100%.

Survival rate after cocultivation = (number of viable explants after cocultivation/total number of infected explants) × 100%.

Proliferation coefficient = (number of explants with a single adventitious bud + number of explants with multiple adventitious buds × 2)/total number of explants.

Adventitious bud germination index = (number of explants with a single adventitious bud + number of explants with multiple adventitious buds)/total number of inoculated seeds.

WPS Office 2023 and SPSS 27.0 were used for the statistical analyses of the data. All the data are presented as the means ± standard deviations (SDs) of three biological replicates. Significant differences were determined using one-way analysis of variance (ANOVA) followed by Duncan’s multiple range test (*p* < 0.05). Graphs were plotted using GraphPad Prism 9.5.1, Adobe Illustrator 2025 and BioGDP.com [38].

## 5. Conclusions

**Figure 5:**
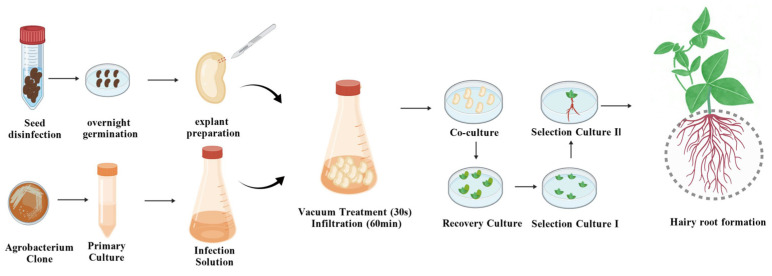
A visualizable hairy root transformation system in cowpea using the *RUBY* reporter.

In summary, an efficient, stable and broadly applicable hairy root genetic transformation system for cowpea was successfully established in this study by screening the optimal genotypes, optimal genotypes, explant types and vector–strain combinations and by optimizing the infection and cocultivation conditions (Figure 5). The system was validated through transformation assays on 86 cowpea genotypes. Two cowpea accessions (JD-0212 and A132) were identified as genotypes with excellent transformation compatibility, cotyledonary nodes with cotyledons were confirmed as the optimal explants for cowpea hairy root transformation, and the combination of *Agrobacterium rhizogenes* strain K599 and the *rbcs-RUBY* vector was selected as the optimal match. The optimal infection conditions included 60 min of infection combined with 30 s of vacuum infiltration, and the optimal cocultivation duration was 4 days. Under these optimal conditions, the maximum transformation efficiency of hairy roots exceeded 82%. Overall, the established system provides a reliable genetic transformation technology for functional genomic research and genetic improvement in cowpea.

## Data Availability

The original contributions presented in this study are included in the article/Supplementary Material. Further inquiries can be directed to the corresponding author.

## Supplementary Materials

The following supporting information can be downloaded at: https://www.mdpi.com/article/10.3390/plants15101560/s1, Table S1: Medium preparation system: This table provides the components, concentrations and preparation procedures of the culture medium, aiming to ensure the reproducibility of the experiment. Table S2: Proliferation coefficient and adventitious shoot induction index in 43 cowpea accessions: This table records the proliferation coefficient and adventitious shoot induction index of 43 cowpea accessions, to screen accessions with high in vitro regeneration capacity. Table S3: Transformation efficiency and tolerance rate of 43 high-frequency in vitro regenerated seedling cowpea genotypes: This table presents the transformation efficiency and tolerance rate of 43 high-regeneration cowpea genotypes, to screen genotypes suitable for genetic transformation. Table S4: Validation of the stability of hairy root genetic transformation system mediated by *rbcs-RUBY* and *bs-EGFP* vectors in 86 cowpea genotypes: This table validates the stability of the hairy root transformation system mediated by two vectors in 86 cowpea genotypes, to provide technical support for subsequent research. Table S5 Information of 86 *Vigna unguiculata* germplasm accessions.
