# Supplementary material for: Development of a High-Efficiency Hairy Root Transformation System for Diverse Cowpea (Vigna unguiculata) Genotypes
Source: Plants (Basel). 2026 May 20;15(10):1560. doi: 10.3390/plants15101560 (PMC13211299; doi:10.3390/plants15101560)
Supplement: Supplementary file 1 [file plants-15-01560-s001.zip › plants-4221687-supplementary.pdf]

Table S1 Medium Preparation System

Medium name	Medium formula
YEB	0.5g/L $MgSO_4 \cdot 7H_2O$ + 1g/L Yeast Extract + 5g/L Beef Extract Powder + 5g/L Tryptone + 5g/L Sucrose + 15g/L Agar Powder
Water Agar Medium	4g/L Agar Powder
Infection Solution	4.4g/L MS + 30g/L Sucrose + 1mg/L PVP + 80mg/L AS + 150mg/L DTT
Co-Cultivation Medium	2.2g/L MS + 30g/L Sucrose + 8g/L Agar Powder + 40mg/L AS + 150mg/L DTT + 0.59g/L MES + 2mg/L Silver Nitrate
Recovery Medium	2.2g/L MS + 30g/L Sucrose + 8g/L Agar Powder + 1mg/L PVP + 2mg/L Silver Nitrate + 0.59g/L MES + 400mg/L Tim
Selection Medium I	2.2g/L MS + 30g/L Sucrose + 8g/L Agar Powder + 1mg/L PVP + 2mg/L Silver Nitrate + 0.59g/L MES + 400mg/L Tim + 2mg/L Basta
Selection Medium II	2.2g/L MS + 30g/L Sucrose + 8g/L Agar Powder + 1mg/L PVP + 2mg/L Silver Nitrate + 0.59g/L MES + 400mg/L Tim + 4mg/L Basta

Note: The pH of YEB is 7.4, while the pH of all other media is 5.8.

Table S2. Proliferation coefficient and adventitious shoot induction index in 43 cowpea accessions

Accession	Proliferation coefficient	Adventitious bud induction index	Accession	Proliferation coefficient	Adventitious bud induction index
**JD-0212**	1.26±0.09	0.90±0.10	**JD-0438**	0.88±0.13	0.75±0.13
**JD-0224**	0.89±0.02	0.77±0.07	**JD-0009**	1.21±0.16	0.94±0.07
**A132**	1.14±0.03	0.95±0.04	**JD-0831**	0.81±0.01	0.75±0.07
**JD-0020**	0.94±0.06	0.75±0.04	**JD-0115**	1.56±0.09	0.94±0.09
**JD-0187**	1.24±0.19	0.80±0.09	**JD-0110**	1.11±0.09	0.85±0.06
**JD-0167**	0.90±0.03	0.77±0.03	**JD-0103**	0.96±0.12	0.72±0.11
**JD-0064**	1.10±0.05	0.73±0.03	**JD-0037**	0.93±0.16	0.77±0.07
**JD-0154**	1.17±0.02	0.83±0.05	**JD-0439**	1.04±0.11	0.76±0.09
**JD-0437**	1.50±0.09	0.80±0.09	**JD-0201**	1.33±0.11	1.00±0.25
**JD-0233**	1.00±0.26	0.74±0.26	**JD-0567**	0.93±0.15	0.78±0.09
**JD-0440**	1.05±0.07	0.74±0.08	**A111G**	1.08±0.07	0.71±0.08
**JD-0039**	1.27±0.12	0.93±0.19	**JD-0032**	1.01±0.13	0.71±0.14
**JD-0159**	0.85±0.03	0.70±0.01	**JD-0181**	0.88±0.01	0.72±0.01
**JD-0804**	1.00±0.18	0.79±0.15	**JD-0234**	0.83±0.04	0.70±0.04
**JD-0105**	0.81±0.12	0.70±0.17	**JD-0825**	1.00±0.15	1.00±0.02
**JD-0807**	1.12±0.10	0.78±0.06	**JD-0200**	1.11±0.21	0.78±0.12
**JD-0104**	0.90±0.13	0.73±0.05	**JD-0823**	0.94±0.22	0.75±0.19
**JD-0312**	1.00±0.05	0.76±0.05	**JD-0441**	0.73±0.06	0.86±0.09
**JD-0140**	0.92±0.00	0.71±0.03	**JD-0100**	1.50±0.02	0.85±0.05
**JD-0483**	0.85±0.26	0.73±0.22	**JD-0018**	0.85±0.05	0.74±0.03
**JD-0153**	1.11±0.14	0.72±0.11	**ZYZ-4950**	1.09±0.15	0.79±0.06
**JD-0114**	1.21±0.13	0.73±0.37			

Note: All data are presented as mean ± standard deviation (SD) of three biological replicates.

Table S3 Transformation efficiency and tolerance rate of 43 high-frequency in vitro regenerated seedling cowpea genotypes

Accession	Stable Transformation efficiency	Tolerance rate	Accession	Stable transformation efficiency	Tolerance rate
**JD-0212**	82.79%±0.03	88.73%±0.01	**JD-0438**	41.38%±0.02	39.38%±0.02
**JD-0224**	73.00%±0.04	86.11%±0.00	**JD-0009**	38.48%±0.01	64.66%±0.02
**A132**	81.79%±0.00	87.71%±0.02	**JD-0831**	38.08%±0.02	67.92%±0.01
**JD-0020**	72.52%±0.04	84.10%±0.01	**JD-0115**	36.94%±0.02	72.85%±0.02
**JD-0187**	71.59%±0.01	62.70%±0.02	**JD-0110**	35.55%±0.02	28.06%±0.01
**JD-0167**	69.52%±0.04	75.88%±0.01	**JD-0103**	33.86%±0.05	52.95%±0.02
**JD-0064**	65.85%±0.02	59.09%±0.03	**JD-0037**	31.30%±0.02	47.40%±0.02
**JD-0154**	65.00%±0.03	41.89%±0.01	**JD-0439**	30.59%±0.03	51.38%±0.02
**JD-0437**	64.85%±0.04	47.27%±0.03	**JD-0201**	29.12%±0.01	84.07%±0.02
**JD-0233**	63.32%±0.01	77.88%±0.01	**JD-0567**	28.59%±0.01	55.52%±0.02
**JD-0440**	55.74%±0.07	58.11%±0.03	**A111G**	25.75%±0.01	92.55%±0.02
**JD-0039**	54.30%±0.01	71.42%±0.03	**JD-0032**	25.40%±0.01	63.92%±0.02
**JD-0159**	54.17%±0.03	78.01%±0.02	**JD-0181**	24.33%±0.02	50.80%±0.02
**JD-0804**	51.73%±0.02	64.63%±0.01	**JD-0234**	23.91%±0.03	74.13%±0.03
**JD-0105**	51.63%±0.01	37.97%±0.01	**JD-0825**	23.77%±0.02	61.50%±0.02
**JD-0807**	51.05%±0.02	73.17%±0.02	**JD-0200**	21.77%±0.01	62.29%±0.03
**JD-0104**	50.53%±0.02	41.86%±0.01	**JD-0823**	18.08%±0.01	83.59%±0.01
**JD-0312**	46.65%±0.04	54.72%±0.01	**JD-0441**	17.98%±0.02	51.68%±0.01
**JD-0140**	46.47%±0.01	53.83%±0.01	**JD-0100**	16.03%±0.01	54.58%±0.01
**JD-0483**	45.89%±0.02	69.52%±0.02	**JD-0018**	15.38%±0.01	60.53%±0.02
**JD-0153**	44.93%±0.00	79.83%±0.02	**ZYZ-4950**	12.72%±0.01	90.87%±0.06
**JD-0114**	43.67%±0.04	68.30%±0.03			

Note: All data in the table are presented as mean ± standard deviation (SD).

Table S4 Validation of the stability of hairy root genetic transformation system mediated by *rbcs-RUBY* and *bs-EGFP* vectors in 86 cowpea genotypes

Accession	*rbcs-RUBY*			*bs-EGFP*		
	Tolerance rate	Transient transformation efficiency	Stable transformation efficiency	Tolerance rate	Transient transformation efficiency	Stable transformation efficiency
**JD-0212**	75.44%±0.12	86.60%±0.02	82.79%±0.03	62.85%±0.08	70.43%±0.11	60.14%±0.01
**A132**	87.71%±0.02	87.54%±0.05	81.79%±0.00	84.21%±0.05	74.47%±0.01	52.55%±0.03
**JD-0006**	82.64%±0.13	88.09%±0.08	76.97%±0.07	67.84%±0.02	78.17%±0.12	73.16%±0.15
**JD-0092**	86.21%±0.09	86.21%±0.09	75.99%±0.10	69.84%±0.07	73.72%±0.12	65.19%±0.15
**JD-0224**	86.11%±0.00	84.52%±0.04	73.00%±0.04	68.93%±0.02	42.28%±0.10	22.99%±0.09
**JD-0020**	84.10%±0.01	83.85%±0.03	72.52%±0.04	79.16%±0.05	43.75%±0.17	29.03%±0.14
**JD-0187**	62.70%±0.02	87.34%±0.10	71.59%±0.01	62.85%±0.08	74.25%±0.06	17.34%±0.10
**JD-0089**	87.67%±0.05	87.67%±0.05	70.21%±0.11	90.62%±0.04	69.57%±0.08	59.53%±0.08
**JD-0167**	75.88%±0.01	85.90%±0.10	69.52%±0.04	75.57%±0.07	70.66%±0.05	53.37%±0.21
**JD-0064**	59.09%±0.03	80.66%±0.15	65.85%±0.02	71.01%±0.10	69.65%±0.05	41.75%±0.07
**JD-0154**	41.89%±0.01	83.79%±0.10	65.00%±0.03	59.82%±0.30	43.86%±0.03	20.32%±0.08
**JD-0437**	47.26%±0.03	81.99%±0.08	64.85%±0.04	66.38%±0.05	76.25%±0.05	36.91%±0.22
**JD-0233**	77.88%±0.01	73.05%±0.07	63.32%±0.01	67.64%±0.18	74.04%±0.04	59.65%±0.18
**JD-0050**	76.55%±0.04	76.55%±0.04	62.61%±0.09	91.75%±0.03	73.88%±0.05	65.26%±0.05
**JD-0065**	78.30%±0.06	78.30%±0.06	62.33%±0.05	94.23%±0.04	78.56%±0.00	48.53%±0.04
**JD-0004**	66.26%±0.14	73.41%±0.04	58.92%±0.08	64.26%±0.02	74.20%±0.03	70.77%±0.07
**JD-0288**	64.53%±0.04	64.53%±0.04	58.00%±0.30	34.30%±0.05	41.38%±0.04	34.10%±0.03
**JD-0814**	61.15%±0.05	61.15%±0.05	57.25%±0.31	40.34%±0.07	47.42%±0.06	35.65%±0.07
**JD-0440**	58.11%±0.03	81.33%±0.09	55.74%±0.07	62.03%±0.29	69.78%±0.02	55.21%±0.16
**JD-0481**	85.57%±0.02	85.57%±0.02	54.91%±0.17	60.46%±0.01	70.50%±0.03	62.20%±0.03
**JD-0039**	56.02%±0.21	69.75%±0.08	54.30%±0.01	77.88%±0.05	33.30%±0.26	24.23%±0.10
**JD-0159**	78.01%±0.02	70.55%±0.03	54.18%±0.03	71.22%±0.10	70.11%±0.04	46.07%±0.07
**XYZ-4947**	56.25%±0.07	56.25%±0.07	53.08%±0.27	85.03%±0.02	77.27%±0.05	70.92%±0.06
**JD-0238**	63.98%±0.04	63.98%±0.04	52.62%±0.39	34.22%±0.09	34.40%±0.09	26.50%±0.09

**JD-0122**	68.83%±0.03	68.83%±0.03	52.52%±0.06	88.79%±0.06	33.08%±0.05	25.18%±0.04
**JD-0804**	64.63%±0.01	67.14%±0.11	51.73%±0.02	78.69%±0.02	68.28%±0.05	58.68%±0.25
**JD-0105**	37.97%±0.01	70.01%±0.15	51.63%±0.01	48.18%±0.03	74.71%±0.02	44.77%±0.08
**JD-0225**	40.95%±0.17	40.95%±0.17	51.07%±0.34	3.09%±0.02	9.53%±0.02	6.25%±0.01
**JD-0807**	73.18%±0.02	70.59%±0.12	51.05%±0.02	65.43%±0.24	69.20%±0.06	55.58%±0.21
**JD-0104**	41.86%±0.01	58.82%±0.03	50.53%±0.02	56.51%±0.04	73.15%±0.06	47.48%±0.02
**JD-0216**	54.65%±0.03	54.65%±0.03	50.37%±0.28	91.88%±0.03	46.72%±0.08	39.43%±0.09
**JD-0270**	70.80%±0.02	70.80%±0.02	50.13%±0.22	92.27%±0.02	48.48%±0.08	42.12%±0.08
**JD-0296**	63.44%±0.07	63.44%±0.07	47.38%±0.27	33.46%±0.04	40.32%±0.03	31.15%±0.05
**JD-0063**	59.65%±0.06	59.65%±0.06	46.80%±0.04	87.36%±0.10	68.33%±0.08	58.44%±0.10
**JD-0312**	54.72%±0.01	51.99%±0.04	46.65%±0.04	60.45%±0.16	69.84%±0.02	52.50%±0.04
**JD-0140**	53.83%±0.01	51.12%±0.01	46.47%±0.01	62.16%±0.07	75.37%±0.09	41.82%±0.12
**JD-0282**	65.83%±0.08	65.83%±0.08	46.24%±0.26	95.19%±0.02	67.82%±0.10	59.71%±0.10
**JD-0818**	52.25%±0.09	52.25%±0.09	46.18%±0.37	17.24%±0.06	24.10%±0.06	17.27%±0.04
**JD-0483**	69.52%±0.02	61.70%±0.14	45.88%±0.02	66.92%±0.07	49.37%±0.05	10.99%±0.08
**JD-0094**	56.82%±0.04	56.82%±0.04	45.27%±0.05	88.65%±0.03	19.15%±0.04	11.46%±0.02
**JD-0153**	79.84%±0.02	61.93%±0.11	44.93%±0.00	65.74%±0.07	47.53%±0.05	36.67%±0.27
**JD-0114**	68.30%±0.03	65.15%±0.11	43.67%±0.04	65.47%±0.09	51.08%±0.07	31.81%±0.19
**JD-0002**	70.51%±0.16	58.04%±0.09	41.52%±0.11	52.94%±0.05	61.94%±0.07	37.86%±0.10
**JD-0438**	39.38%±0.02	62.23%±0.15	41.38%±0.02	43.98%±0.02	68.51%±0.01	61.70%±0.25
**JD-0009**	64.66%±0.02	55.75%±0.11	38.49%±0.01	76.48%±0.03	19.89%±0.13	32.01%±0.21
**JD-0831**	67.92%±0.01	55.68%±0.08	38.08%±0.02	65.86%±0.08	51.34%±0.08	24.07%±0.02
**JD-0115**	72.85%±0.02	55.66%±0.12	36.94%±0.02	65.56%±0.20	68.78%±0.04	38.19%±0.10
**JD-0019**	52.39%±0.07	52.39%±0.07	36.56%±0.17	95.40%±0.02	70.86%±0.08	60.92%±0.10
**JD-0110**	28.06%±0.01	53.03%±0.12	35.55%±0.02	22.14%±0.07	73.82%±0.02	33.75%±0.29
**JD-0124**	47.19%±0.15	47.19%±0.15	34.94%±0.33	83.06%±0.01	68.17%±0.02	57.06%±0.02
**JD-0103**	52.95%±0.02	54.31%±0.15	33.86%±0.05	65.74%±0.07	57.28%±0.25	12.65%±0.10
**JD-0143**	59.64%±0.11	59.64%±0.11	32.13%±0.07	92.47%±0.02	71.90%±0.08	63.13%±0.09
**JD-0074**	41.94%±0.06	41.94%±0.06	31.85%±0.03	14.19%±0.02	19.89%±0.02	12.80%±0.02

**JD-0220**	43.18%±0.10	43.18%±0.10	31.43%±0.24	87.13%±0.01	71.80%±0.09	62.57%±0.11
**JD-0037**	47.40%±0.02	47.62%±0.05	31.30%±0.02	62.26%±0.09	66.65%±0.43	32.77%±0.28
**JD-0439**	51.38%±0.02	45.96%±0.04	30.59%±0.03	66.28%±0.05	75.77%±0.03	39.76%±0.17
**JD-0021**	41.65%±0.07	41.65%±0.07	30.36%±0.05	14.44%±0.03	27.19%±0.08	18.17%±0.06
**JD-0176**	49.56%±0.04	49.56%±0.04	30.07%±0.38	92.06%±0.04	75.89%±0.10	64.92%±0.10
**JD-0813**	32.39%±0.04	32.39%±0.04	29.46%±0.24	79.57%±0.06	73.55%±0.06	70.13%±0.09
**JD-0201**	84.07%±0.02	45.09%±0.07	29.12%±0.01	73.35%±0.09	22.05%±0.16	30.87%±0.22
**JD-0567**	55.52%±0.02	44.52%±0.07	28.59%±0.01	64.62%±0.09	66.34%±0.12	28.63%±0.17
**JD-0914**	84.05%±0.03	84.05%±0.03	27.40%±0.24	94.22%±0.03	79.38%±0.02	73.26%±0.02
**JD-0292**	67.91%±0.24	67.91%±0.24	27.40%±0.07	28.90%±0.06	36.07%±0.09	27.91%±0.05
**JD-0808**	90.36%±0.05	90.36%±0.05	27.13%±0.08	72.23%±0.01	65.82%±0.01	53.12%±0.07
**A111G**	92.55%±0.02	34.12%±0.02	25.75%±0.01	87.84%±0.06	80.70%±0.02	58.31%±0.01
**JD-0032**	63.92%±0.02	39.53%±0.03	25.40%±0.01	79.32%±0.01	15.58%±0.01	4.04%±0.03
**JD-0181**	50.80%±0.02	48.03%±0.16	24.34%±0.02	62.26%±0.09	74.99%±0.04	14.70%±0.12
**JD-0234**	74.13%±0.03	42.01%±0.15	23.91%±0.03	77.35%±0.04	27.99%±0.09	12.54%±0.05
**JD-0825**	61.50%±0.02	41.08%±0.17	23.77%±0.02	77.91%±0.02	60.91%±0.06	36.38%±0.07
**JD-0279**	59.31%±0.11	59.31%±0.11	22.07%±0.05	24.79%±0.05	31.70%±0.05	23.40%±0.05
**JD-0285**	42.03%±0.05	42.03%±0.05	21.87%±0.09	91.93%±0.03	75.60%±0.06	67.59%±0.07
**JD-0200**	46.85%±0.12	38.12%±0.07	21.76%±0.01	64.66%±0.13	46.65%±0.12	37.24%±0.30
**JD-0247**	91.02%±0.07	91.02%±0.07	21.64%±0.16	94.87%±0.03	76.32%±0.05	68.52%±0.06
**JD-0010**	48.27%±0.27	35.09%±0.04	21.28%±0.05	92.09%±0.01	55.60%±0.10	46.86%±0.13
**JD-0577**	55.47%±0.06	55.47%±0.06	20.27%±0.04	92.67%±0.03	72.22%±0.05	71.52%±0.12
**JD-0823**	83.59%±0.01	38.27%±0.14	18.08%±0.01	79.56%±0.00	26.43%±0.12	19.93%±0.14
**JD-0441**	51.68%±0.01	32.03%±0.12	17.98%±0.02	64.62%±0.09	77.43%±0.05	31.03%±0.17
**JD-0915**	67.23%±0.29	67.23%±0.29	16.19%±0.10	69.08%±0.07	58.88%±0.26	51.65%±0.25
**JD-0100**	54.58%±0.01	36.03%±0.09	16.03%±0.01	66.28%±0.05	73.56%±0.03	41.32%±0.04
**XYZ-4951**	38.42%±0.09	38.42%±0.09	15.79%±0.13	89.55%±0.01	59.16%±0.11	52.02%±0.11
**JD-0213**	13.71%±0.07	13.71%±0.07	15.65%±0.08	95.12%±0.03	41.31%±0.05	34.29%±0.06
**JD-0017**	26.41%±0.03	26.41%±0.03	15.45%±0.07	92.39%±0.01	44.41%±0.05	35.59%±0.03

**JD-0018**	60.53%±0.02	32.37%±0.07	15.38%±0.01	67.21%±0.29	51.31%±0.20	50.61%±0.10
**ZYZ-4950**	90.87%±0.06	29.14%±0.01	12.72%±0.01	65.96%±0.08	50.13%±0.07	28.90%±0.09
**JD-0810**	64.10%±0.05	64.10%±0.05	11.33%±0.08	93.92%±0.03	84.76%±0.06	72.75%±0.08
**JD-0302**	31.65%±0.04	31.65%±0.04	9.17%±0.03	94.12%±0.04	56.77%±0.14	50.70%±0.13

Note: All data in the table are presented as mean ± standard deviation (SD).The validation of the hairy root genetic transformation system stability involved 86 dual-vector verification tests, which covered 43 cowpea accessions with high-frequency *in vitro* regeneration.

Table S5 Information of 86 *Vigna unguiculata* germplasm accessions

Accession	Collection site	Pod type	Resource type
**JD-0212**	Liaoning	Short-pod type	Local variety
**A132**	Guangdong	Short-pod type	Local variety
**JD-0006**	Beijing	Long-pod type	Local variety
**JD-0092**	Beijing	Long-pod type	Local variety
**JD-0224**	Tianjin	Long-pod type	Local variety
**JD-0020**	Guangdong	Short-pod type	Local variety
**JD-0187**	Anhui	Long-pod type	Local variety
**JD-0089**	Anhui	Long-pod type	Local variety
**JD-0167**	Hubei	Short-pod type	Commercial variety
**JD-0064**	Hubei	Long-pod type	Commercial variety
**JD-0154**	Guangdong	Long-pod type	Commercial variety
**JD-0437**	Sichuan	Long-pod type	Local variety
**JD-0233**	Hubei	Long-pod type	Local variety
**JD-0050**	Hubei	Short-pod type	Local variety
**JD-0065**	Liaoning	Long-pod type	Local variety
**JD-0004**	Guangdong	Long-pod type	Local variety
**JD-0288**	Tianjin	Short-pod type	Local variety
**JD-0814**	Hubei	Long-pod type	Local variety
**JD-0440**	Hubei	Long-pod type	Local variety
**JD-0481**	Guangdong	Long-pod type	Commercial variety
**JD-0039**	Fujian	Long-pod type	Commercial variety
**JD-0159**	Ningxia	Long-pod type	Commercial variety
**XYZ-4947**	Anhui	Long-pod type	Local variety
**JD-0238**	Hubei	Short-pod type	Local variety
**JD-0122**	Beijing	Long-pod type	Local variety
**JD-0804**	Liaoning	Long-pod type	Local variety
**JD-0105**	USA	Long-pod type	Local variety
**JD-0225**	Sichuan	Long-pod type	Local variety
**JD-0807**	Hubei	Long-pod type	Local variety
**JD-0104**	Jiangsu	Long-pod type	Local variety

**JD-0216**	Fujian	Long-pod type	Commercial variety
**JD-0270**	Hubei	Short-pod type	Commercial variety
**JD-0296**	Jiangxi	Long-pod type	Commercial variety
**JD-0063**	Jiangxi	Long-pod type	Local variety
**JD-0312**	Tianjin	Long-pod type	Local variety
**JD-0140**	Guangdong	Long-pod type	Local variety
**JD-0282**	Hubei	Long-pod type	Commercial variety
**JD-0818**	Hubei	Long-pod type	Commercial variety
**JD-0483**	Anhui	Long-pod type	Commercial variety
**JD-0094**	Hubei	Short-pod type	Local variety
**JD-0153**	Hubei	Long-pod type	Local variety
**JD-0114**	Jiangsu	Long-pod type	Local variety
**JD-0002**	Ningxia	Short-pod type	Commercial variety
**JD-0438**	Sichuan	Short-pod type	Commercial variety
**JD-0009**	Hubei	Short-pod type	Commercial variety
**JD-0831**	Guangdong	Long-pod type	Commercial variety
**JD-0115**	Hubei	Long-pod type	Local variety
**JD-0019**	Hubei	Short-pod type	Local variety
**JD-0110**	Hubei	Short-pod type	Local variety
**JD-0124**	Jiangxi	Short-pod type	Local variety
**JD-0103**	Hubei	Long-pod type	Local variety
**JD-0143**	Hubei	Long-pod type	Local variety
**JD-0074**	Guangdong	Short-pod type	Local variety
**JD-0220**	Hubei	Long-pod type	Local variety
**JD-0037**	Hubei	Long-pod type	Commercial variety
**JD-0439**	Hubei	Long-pod type	Commercial variety
**JD-0021**	Hubei	Long-pod type	Commercial variety
**JD-0176**	Guangdong	Long-pod type	Commercial variety
**JD-0813**	Hubei	Long-pod type	Commercial variety
**JD-0201**	Hubei	Long-pod type	Local variety
**JD-0567**	Jiangsu	Long-pod type	Local variety
**JD-0914**	Hubei	Long-pod type	Local variety
**JD-0292**	Hubei	Long-pod type	Local variety
**JD-0808**	Jiangsu	Long-pod type	Local variety
**A111G**	Hubei	Long-pod type	Commercial variety
**JD-0032**	Jiangxi	Short-pod type	Commercial variety
**JD-0181**	Hubei	Short-pod type	Commercial variety
**JD-0234**	Jiangsu	Short-pod type	Local variety
**JD-0825**	Hubei	Long-pod type	Local variety
**JD-0279**	Anhui	Long-pod type	Local variety
**JD-0285**	Hubei	Short-pod type	Commercial variety
**JD-0200**	Guangdong	Long-pod type	Commercial variety
**JD-0247**	Hubei	Short-pod type	Commercial variety
**JD-0010**	Jiangxi	Long-pod type	Local variety

**JD-0577**	Hubei	Long-pod type	Local variety
**JD-0823**	Jiangxi	Long-pod type	Local variety
**JD-0441**	Jiangsu	Short-pod type	Local variety
**JD-0915**	Jiangxi	Long-pod type	Local variety
**JD-0100**	Hubei	Long-pod type	Commercial variety
**XYZ-4951**	Anhui	Long-pod type	Commercial variety
**JD-0213**	Jiangxi	Long-pod type	Commercial variety
**JD-0017**	Guangdong	Long-pod type	Local variety
**JD-0018**	Jiangxi	Long-pod type	Local variety
**ZYZ-4950**	Hubei	Short-pod type	Local variety
**JD-0810**	Jiangxi	Short-pod type	Local variety
**JD-0302**	Hubei	Long-pod type	Local variety

Note: Long-pod type: pod length > 30 cm; Short-pod type: pod length ≤ 30 cm.
